# Accuracy of Smartwatches in the Detection of Atrial Fibrillation: A Systematic Review and Diagnostic Meta-Analysis

**DOI:** 10.1016/j.jacadv.2025.102133

**Published:** 2025-11-03

**Authors:** Nelson Barrera, Maria Solorzano, Yomary Jimenez, Yevhen Kushnir, Francisco Gallegos-Koyner, Guilherme Dagostin de Carvalho

**Affiliations:** a SBH Health System, Department of Internal Medicine, City University of New York School of Medicine, Bronx, New York, USA; b Department of Internal Medicine, Division of Cardiovascular Medicine, University of Florida Health, Gainesville, Florida, USA; c Dante Pazzanese Institute of Cardiology, Department of Cardiac Arrhythmias and Electrophysiology, São Paulo, Brazil

**Keywords:** atrial arrhythmia, atrial fibrillation, electrocardiography, smartwatch technology, stroke

## Abstract

**Background:**

Atrial fibrillation (AF) is the most prevalent arrhythmia and a significant risk factor for stroke and heart disease, making early diagnosis crucial for prevention. Although several smartwatches can detect AF, their accuracy varies, and there is limited information regarding their effectiveness.

**Objectives:**

The purpose of this study was to analyze the overall accuracy of current available smartwatches in identifying AF.

**Methods:**

A comprehensive systematic literature search was performed across major databases for articles published until January 2025. The specificity, sensitivity, and area under the curve (AUC). Statistical analysis was conducted using R software. The review protocol was registered in PROSPERO (CRD420251007932).

**Results:**

Twenty-six studies were included in the study, including 17,349 patients. Overall sensitivity was 95% (95% CI: 92%-97%; I^2^ = 95%) and specificity 97% (95% CI: 94%-98%; I^2^ = 99%), with a pooled AUC of 0.97 (95% CI: 0.96%-0.99%). Device performance varied, with the Apple Watch achieving 94% sensitivity (95% CI: 89%-96%; I^2^ = 71%) and 97% specificity (95% CI: 93%-99%; I^2^ = 87%), Samsung devices yielding 97% sensitivity (95% CI: 92%-99%; I^2^ = 92%) and 96% specificity (95% CI: 91%-98%; I^2^ = 92%), and the Withings Scan Watch showing 89% sensitivity (95% CI: 75%-96%; I^2^ = 94%) and 95% specificity (95% CI: 88%-99%; I^2^ = 93%). Accuracy was comparable between photoplethysmography and electrocardiogram-based models.

**Conclusions:**

Smartwatches possess excellent diagnostic accuracy for AF detection and may, therefore, represent a suitable and fitting option for patients.

Atrial fibrillation (AF) is one of the most common cardiac arrhythmias and a major risk factor for stroke, affecting over 52.5 million people worldwide as of 2021—a 137% increase since 1990.^1^ In this case, oral anticoagulation can safely prevent AF-related strokes when indicated.^2^^,^^3^ While early detection of subclinical AF, along with the initiation of oral anticoagulation, has been associated with a decrease in stroke rates, it also correlates with an increase in bleeding events. However, these findings may not be applicable to patients with low-burden disease.^4^ In contrast, the LOOP (Implantable loop recorder detection of atrial fibrillation to prevent stroke) trial, which randomized patients to receive either implantable loop recorder screening or usual care, found no statistically significant reduction in risk of stroke or systemic arterial embolism despite an increase in AF detection.^5^ These conflicting results underscore the importance of individual risk stratification and personalized decision-making regarding screening.

Traditionally, an electrocardiogram (ECG) has been the gold standard for AF diagnosis, identifying irregular electrical conduction in the left atrium. However, advances in digital health technology have introduced smartwatches equipped with artificial intelligence–driven algorithms and sensor technologies, such as photoplethysmography (PPG) and ECG, offering a noninvasive and accessible means of AF detection.^6^ Despite their potential, interpreting smartwatch-generated data remain challenging due to variations in technology and algorithms across different devices.^7^

Recently, several smartwatches received approval from the Food and Drug Administration and Conformité Européenne, with the Apple Watch being the pioneer by obtaining its approval in 2018. This device is equipped with a single-lead ECG sensor that can record a 30-second ECG and detect AF. Additionally, the AF detection algorithm was Food and Drug Administration-cleared, enabling passive monitoring of irregular heart rhythms using PPG with high accuracy.^8^ The Withings ScanWatch followed in 2021 with similar capabilities, using an integrated ECG sensor and PPG technology.^9^ More recently, Samsung’s smartwatch received approval for its Irregular Heart Rhythm Notification system, which employs ECG technology and machine learning to detect AF and even sleep apnea.

Given the variability in smartwatch efficacy for AF detection, it is essential to consolidate current evidence on their diagnostic accuracy. Previous studies were limited by design and sample size, and new devices have since entered the market.^10^^,^^11^ A recent review by Shahid et al reported high sensitivity and specificity for AF detection using the Apple Watch ECG.^8^ This review aims to provide a comprehensive assessment of smartwatches' effectiveness in AF detection, compare the diagnostic performance of different smartwatch models and underlying technologies, and evaluate their role in AF detection following secondary events such as stroke or transient ischemic attacks.

## Methods

### Protocol and registration

This systematic review followed the PRISMA 2020 (Preferred Reporting Items for Systematic Reviews and Meta-Analyses) guidelines.^12^ The review protocol was registered in PROSPERO (International Prospective Register of Systematic Reviews) (CRD420251007932).

### Literature search

Two independent reviewers (N.B. and M.S.) conducted the literature search using a two-step approach. First, they developed a comprehensive search strategy tailored for 3 electronic databases: ScienceDirect, PubMed, and CENTRAL. The keywords utilized for the electronic search on PubMed included ((Smartwatch OR Watch) AND (“Atrial Fibrillation” OR “Arrhythmia” OR “Cardiac Arrhythmia”) AND (“Diagnostic accuracy” OR “Accuracy”)). These keywords were modified for each database to maximize relevant results.

In the second step, the reviewers manually screened the reference lists of identified studies to capture additional relevant articles. Additionally, they searched ClinicalTrials.gov for completed trials with results that had not yet been published.

### Eligibility criteria

After retrieving articles from databases and registries, the authors applied prespecified eligibility criteria to evaluate each article for inclusion in the review. The selected articles met the following inclusion criteria: 1) published in English; 2) included patients diagnosed with AF; 3) investigated the use of any type of smartwatch technology for detecting AF; 4) reported outcomes related to the diagnostic accuracy of smartwatch technology in detecting AF and differentiating it from other heart arrhythmias; and 5) were designed as clinical trials, case-control studies, or diagnostic accuracy studies of smartwatch technologies.

Articles were excluded from the review based on the following criteria: 1) did not include patients with AF; 2) evaluated the efficacy of wearable devices other than smartwatches; 3) did not report outcomes related to the diagnostic accuracy of smartwatch technology in detecting AF; 4) were secondary studies, such as systematic reviews, narrative reviews, or meta-analyses; and 5) were case reports or case series.

### Data extraction

Two reviewers independently conducted the data extraction for this review. In instances where consensus was lacking, the reviewers discussed the discrepancies until they reached an agreement. Following PRISMA guidelines, the authors meticulously reviewed all references obtained throughout various phases of the process before data extraction. The initial phase involved screening abstracts for relevance, leading to the elimination of irrelevant articles. The full texts of the remaining articles were then sought and assessed for eligibility criteria. Articles that met our inclusion criteria were subsequently used for data extraction. The information extracted from each article included the author identification, study setting, study design, sample size, type of smartwatch utilized, underlying technology, comparator, participant age, and the reference standard used in the study.

### Quality assessment

We employed the Quality Assessment of Diagnostic Accuracy Studies (QUADAS-2) tool to evaluate the methodological quality of the included diagnostic accuracy studies. This tool facilitated the assessment of study quality based on criteria such as patient selection, reference standard, index test, and study flow and timing. The results of the risk of bias assessment were then illustrated using a summary graph.

### Statistical analysis

Statistical analysis was performed using R version 4.2, utilizing the Mada package to conduct a meta-analysis of sensitivity, specificity, positive predictive value (PPV), and negative predictive value (NPV) derived from 2 × 2 contingency tables from each study. In the outcomes, a true positive was considered a diagnosis of AF by the smartwatch, confirmed by the reference standard. A true negative was considered a diagnosis of normal sinus rhythm by the smartwatch, which was confirmed by the reference standard. A false positive was considered a diagnosis of AF by the smartwatch, confirmed to be normal sinus rhythm by the reference standard, and a false negative was considered a diagnosis of normal sinus rhythm by the smartwatch, confirmed to be AF by the reference standard. Four values were then used to calculate the sensitivity, specificity, PPV, NPV, the diagnostic OR (DOR), and the area under the curve (AUC). A bivariate random-effects regression model was used to aggregate sensitivity and specificity, generating summary receiver-operating characteristic (SROC) curves along with their respective areas under the curve. In addition to the univariate analysis, we conducted a random-effect meta-analysis of the DOR using the DerSimonian-Laird estimator on log-transformed DORs. The results were presented in forest plots and graphs. Heterogeneity was quantified using the I^2^ statistic, with values categorized as follows: 0% to 25% indicating insignificant heterogeneity, 26% to 50% indicating low heterogeneity, 51% to 75% indicating moderate heterogeneity, and >75% indicating high heterogeneity. A meta-regression analysis was conducted to assess the impact of different smartwatch types, baseline technological parameters, and sample size on the odds of accurately diagnosing AF with smartwatch devices. Additionally, a prespecified subgroup analysis by device generation was performed. To assess publication bias, we conducted a Deeks' funnel plot asymmetry test using a meta-regression approach, in which the natural logarithm of the DOR (log DOR) was regressed against the inverse square root of the effective sample size (1/√ESS), as described in previously published methods for diagnostic test accuracy meta-analyses.^13^ A statistically significant nonzero slope was considered indicative of potential small-study effects or publication bias. We also performed a leave-one-out sensitivity analysis by iteratively removing one study at a time and recalculating the pooled estimates of sensitivity, specificity, PPV, and NPV using random-effects models. This analysis was conducted to evaluate the robustness and stability of the summary estimates with respect to individual study influence.

## Results

### Search results

**Figure 1:**
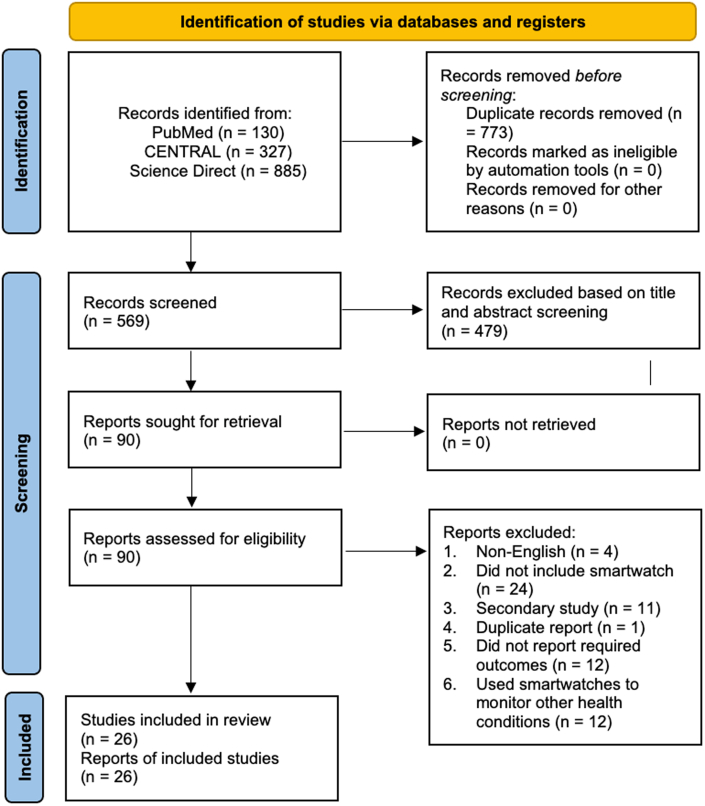
**PRISMA Flow Diagram of Study Selection Process** A total of 1,342 records were identified through database searches. After removing duplicates and ineligible records, 90 full-text articles were assessed for eligibility. Of these, 26 studies met the inclusion criteria and were included in the final review. Reasons for exclusion are detailed at each stage. PRISMA = Preferred Reporting Items for Systematic Reviews and Meta-Analyses.

Our literature search yielded 1,343 articles, among which 773 were duplicates and thus were excluded from the study. This led to 569 abstracts being screened. After the abstract screening, 479 were deemed irrelevant to the topic of study and were thus excluded from the study, and 90 articles were sought for retrieval. All the 90 full texts were retrieved and assessed for eligibility for our review. After the eligibility assessment, 26 articles were included in the review, and 64 were excluded. The following were the reasons for the exclusion from the review: four were not published in English, 24 did not include a smartwatch as one of the interventions, 11 were secondary studies, one was a duplicate article from the same study, 12 did not report any of the required outcomes, and 12 used smartwatches to analyze other health conditions. A PRISMA diagram summarizing the search strategy is presented in Figure 1.

### Characteristics of the included studies

This review included 26 diagnostic accuracy studies conducted across various countries, including Japan, the United States, Switzerland, Germany, Norway, France, China, Turkey, the Netherlands, Belgium, and Taiwan. These studies collectively analyzed data from 17,379 patients, with a pooled median age of 66.5 (64.0-68.0) years, with a pooled prevalence of AF of 111 per 1,000 patients, which corresponds to an overall prevalence rate of approximately 11%. The detailed characteristics of the included studies are presented in Table 1.

**Table 1:** Characteristics of the Studies Included in the Meta-Analysis

First Author, Year	Setting	Study Design	Type of Watch	Participants	Age (Median/Mean) (y)	Sample Size	CHA_2_DS_2-_VASc	Technology	AF Prevalence (%)	Low Quality Recordings (%)	Reference Standard
Wouters et al, 2025^14^	Belgium	Prospective cohort	Apple Watch	Adult patients with either sinus rhythm or AF.	69 (61-77)	122	0-6	Single-lead ECG	30 (24.6)	10%	12-lead ECG
Wasserlauf et al, 2023^15^	USA	Prospective cohort	Apple Watch	Patients with an ICM, AF lasting >1 hour within 90 days of enrollment to the study.	65.4 ± 12.2	30	NR	PPG	11 (37)	NR	ICM or CIED
Müller et al, 2024^16^	Norway	Cross-sectional study	Apple Watch	Adult patients who had undergone left open-heart valve surgery.	68 ± 9.9	93	NR	Single-lead ECG	18 (19.3)	6.4%	12-lead ECG
Ding et al, 2023^32^	USA	Randomized controlled trial	Pulse watch	Adult patients aged ≥50 y with a history of TIA or stroke.	65	57	NR	PPG	6 (6.67)	NR	ECG patch
Manhart et al, 2023^17^	Switzerland	Prospective diagnostic study	Apple Watch	Adult patients scheduled for catheter ablation.	67 (58-75)	201	2 (1-3)	ECG	62 (31)	21%	12-lead ECG
			Samsung Galaxy Watch 6								
			Withings Scan Watch								
			Fitbit Sense								
Campo et al, 2022^18^	France	Multicenter study	Scan Watch	Adult patients with either AF or sinus rhythm.	67.7 ± 14.8	262	NR	ECG	100 (38)	6.9%	12-lead ECG
Ploux et al, 2022^19^	France	Prospective cohort	Apple Watch Series 4	Adult patients with or without a history of cardiac diseases.	66 ± 6	260	NR	ECG	49 (19)	NR	12-lead ECG
Abu-Alrub et al, 2022^20^	France	Prospective cohort	Apple Watch Series 5	Patients who had AF either preablation or postablation.	62 ± 7	200	NR	ECG	100 (50)	12.6%	12-lead ECG
			Samsung Galaxy Watch active 3								
			Withings move ECG								
Pepplinkhuizen et al, 2022^21^	Netherlands	Prospective cohort	Apple Watch 6	Adult AF patients are scheduled for elective electrical cardioversion.	67.1 ± 12.3	74	3.0 [1.75–4.0]	ECG	65 (50.3)	24.8%	12-lead ECG
Alnasser et al, 2023^22^	Saudi Arabia.	Prospective cohort	Apple Watch 6	Adult patients attending a cardiac outpatient clinic.	56.43 ± 16.30	469	NR	ECG	34 (7.20)	NR	12-lead ECG
Racine et al, 2022^23^	France	Prospective cohort	Apple Watch 6	Adult hospitalized patients.	66	734	NR	ECG	154 (21)	21%	12-lead ECG
Scholsten et al, 2022^9^	Netherlands	Prospective cohort	Withings	Adult patients admitted for electrical cardioversion for AF or atrial flutter (AFL).	70 ± 10	220	NR	ECG	187 (45)	21%	12-lead ECG
			Apple Watch				NR	ECG		NR	
Pasli et al, 2024^24^	Turkey	Prospective cohort	Apple Watch 7	Patients presenting to the emergency department requiring immediate care.	65 ± 16.3 (males), 68 ± 17.9 (females)	721	NR	ECG	180 (25)	2.9%	12-lead ECG
Pan et al, 2025^25^	China	Prospective cohort	Amazfit Health Smartwatch	Patients with AF who were admitted in the in-patient wards for catheter ablation. Also, paroxysmal or persistent AF in the outpatient department.	65.4 ± 8.5	78	NR	ECG and PPG	7.9%	45.4% for PPG and 12.8% for ECG	Holter monitor
Nonoguchi et al, 2022^26^	Japan	Prospective cohort	Seikon Epson smartwatch	Patients who were assessed for AF in the outpatient department.	66 ± 12 (high-risk), 67 ± 12 (known-AF group)	286	1.9 ± 1 (high risk) 1.8 ± 12 (AF group)	PWM	51 (18)	NR	Telemetry ECG
Badertscher et al, 2022^27^	Switzerland	Prospective cohort	Withings smartwatch	Patients who presented to the cardiology department.	67 (54-76)	319	NR	ECG	34 (11)	14%	12-lead ECG
Avram et al, 2021^28^	USA	Prospective cohort	Samsung	Patients with a self-reported diagnosis of AF or risk of developing A-fib.	62.61 ± 11.60	204	0-7	PPG	107 (53)	1%	ECG patch
Bumgarner et al, 2018^29^	USA	Prospective cohort	Apple Watch	Adult patients aged 18-90 y with a diagnosis of AF who were willing to wear the watch before and after the cardioversion.	68 ± 11	100	NR	ECG	91 (54)	33.7	12-lead ECG
Rajakariar et al, 2020^30^	Australia	Prospective cohort	Kardiaband and Apple Watch	Adult patients admitted to the medical, cardiac or intensive care units.	64 ± 17 (sinus rhythm) and 76 ± 11 (AF)	218	2 (SR) 3 (AF)		38 (19)	19.5%	12-lead ECG
Wasserlauf et al, 2019^31^	USA	Prospective cohort	Kardiaban and Apple Watch 2	Adult patients with previously implanted insertable cardiac monitors and a history of paroxysmal AF.	72.1 ± 7.2	26	3 ± 1.3	PPG	18 (75)	NR	ECG
Ding et al, 2019^32^	USA	Prospective cohort	Simband 2	Adult participants aged 21 y or more.	70.6 ± 8	40	2.6 ± 1.3	PPG and ECG	63 (20)	87.6%	Holter monitor
Tison et al, 2018^33^	USA	Prospective cohort	Apple Watch	Adult participants.	55.7 ± 14.2 (AF), 41.5 ± 11.9 (sinus rhythm)	9,750	NR	PPG	64 (4)	NR	12-lead ECG
Perez et al, 2019^34^	USA	Prospective cohort	Apple Watch	Adult participants aged 22 y or more.	57 ± 15	2,161	Score ≥2 (13%)	PPG	153 (34)	3.8%	ECG patch
Bashar et al, 2019^35^	USA	Prospective cohort	Simband	Adult patients with either AF or sinus rhythm.	NR.	46	NR	PPG	55 (17.5)	87%	Holter monitors ECG data
Dörr et al, 2019^36^	Germany and Switzerland	Case-control study.	Samsung Gear fit 2	Adult patients without pacemakers or implanted defibrillators.	76.4 ± 9.5	508	NR	PPG	237 (47)	NR	Cardiologists ECG diagnoses
Chang et al., 2022^49^	Taiwan	Prospective	Garming Forerunner 945	Patients ≥19 y.	66.1 ± 12.6	200	NR	PPG	116 (56)	66%	Holter

AF = atrial fibrillation; CIED = cardiac implanted electronic device; ECG = electrocardiogram; ICM = implanted cardiac monitor; NR = not reporte; PPG = photoplethysmography; PWM = pulse wave monitor; TIA = transient ischemic attackd.

### Quality assessment

**Figure 2:**
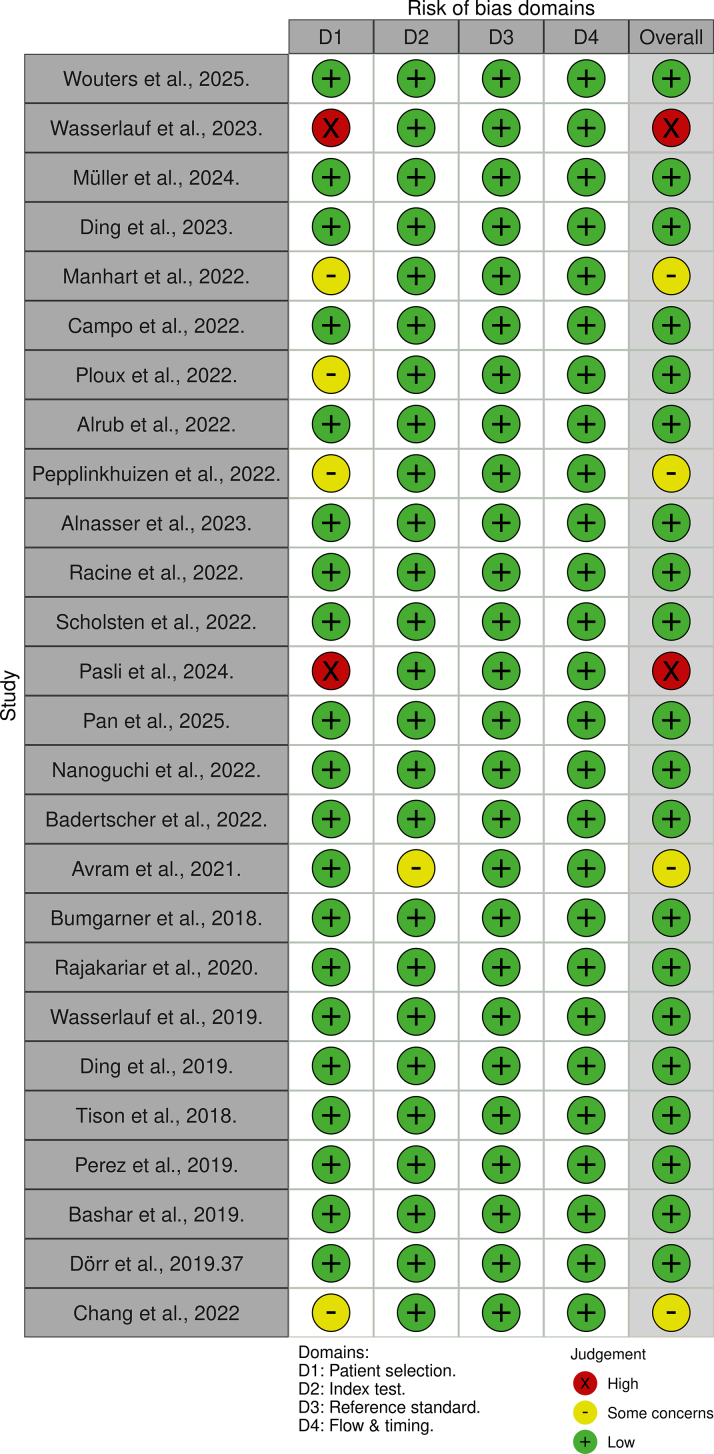
**Risk of Bias Assessment Using QUADAS-2 Tool** This figure presents the risk of bias assessment for the 26 included studies using the QUADAS-2 framework. Most studies demonstrated low risk of bias across domains, though a few showed methodological limitations in patient selection and index test conduct. The overall risk of bias is summarized in the final column. QUADAS = Quality Assessment of Diagnostic Accuracy Studies.

Most of the included studies demonstrated good methodological quality. However, studies with a high risk of bias primarily included samples consisting solely of patients with AF, which does not accurately represent a real-world clinical setting. Additionally, some studies did not specify whether the interpretation of smartwatch data was conducted in a blinded manner, raising concerns about potential bias Figure 2. In the included studies, publication bias was assessed using a Deeks’ funnel plot (Supplemental Figure 1). The regression slope was statistically significant (β = 3.80; 95% CI: 0.02%-7.57%; *P* = 0.048), suggesting the presence of small study effects and potential publication bias; also, the effect size explained 7.8% of the attribute heterogeneity (Supplemental Table 1). Our leave-one analysis shows that none of the included studies significantly impacted our results (Supplemental Figures 2 to 5). The GRADE (Good Research for Comparative Effectiveness) framework indicates that the quality of evidence was rated as moderate (Supplemental Figure 6). While the risk of bias and indirectness were not deemed serious for the majority of studies, we opted for a downgrade due to serious inconsistency, characterized by substantial heterogeneity (I^2^ > 95%) among the studies analyzed, which was explained by sample size, publication bias, and difference in device generation.

### Meta-analysis outcomes

#### Sensitivity

**Figure 3:**
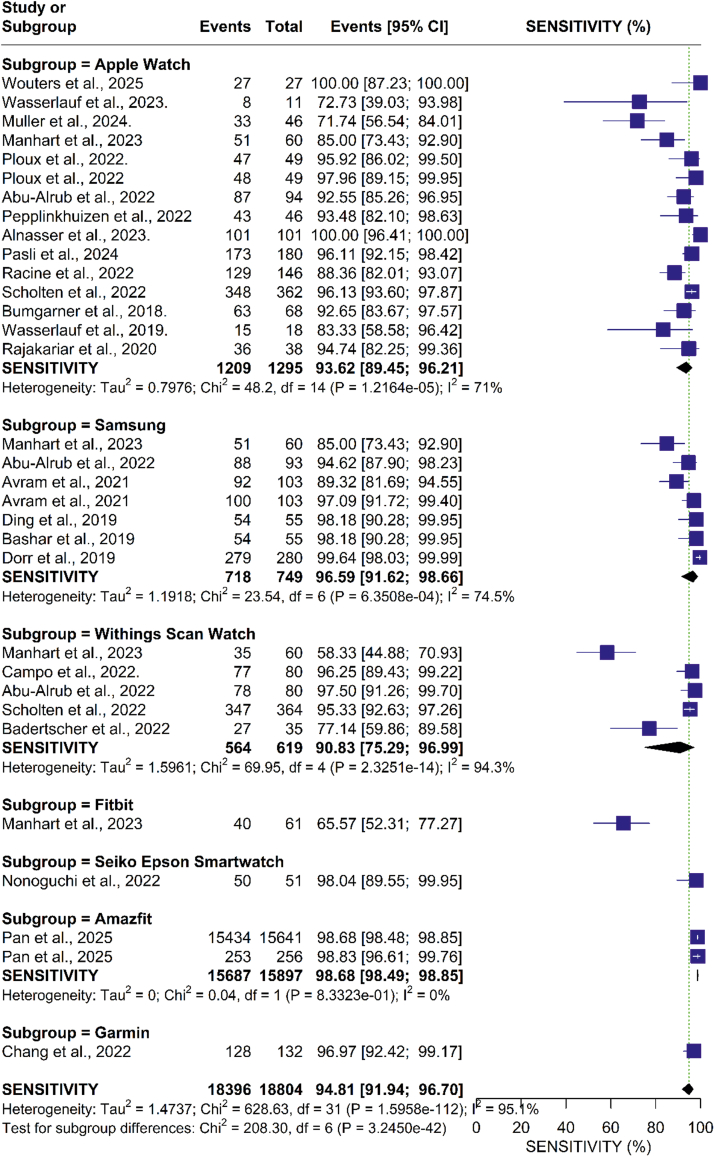
**Forest Plot of Sensitivity for Device Subgroup Analysis** The forest plot presents the pooled sensitivity estimates of smartwatches for detecting atrial fibrillation, stratified by device brand. The pooled sensitivity across all studies was 94.81% (95% CI: 91.94% to 96.70%).

Our analysis showed that smartwatches had a pooled sensitivity of 94.81% (95% CI: 91.94%-96.70%; I = 95%) for detecting AF. A subgroup analysis based on smartwatch type revealed that the Apple Watch had a sensitivity of 93.62% (95% CI: 89.45%-96.21%; I = 71%), Samsung 96.59% (95% CI: 91.62%-98.66%; I = 74%), Withing’s 88.58% (95% CI: 67.19%-96.71%; I = 94%), Fitbit 65.57% (95% CI: 52.31%-77.27%), Scan Watch 96.25% (95% CI: 89.43%-99.22), Seiko Epson Smartwatch 98.04% (95% CI: 89.55%-99.95%), and Amazfit 98.68% (95% CI: 98.49%-98.85%; I = 0%), Garmin Forerunner 945 96.97% (95% CI: 92.42%-99.17%). A forest plot summarizing these results is presented in Figure 3.

#### Specificity

**Figure 4:**
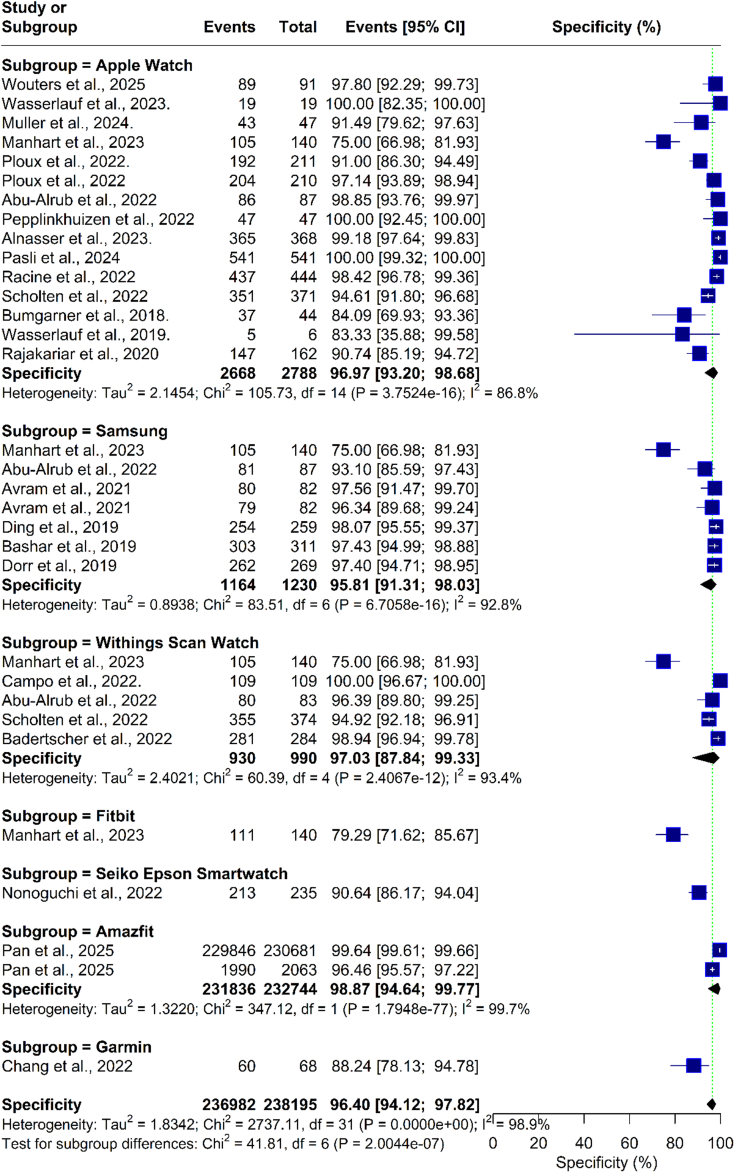
**Forest Plot of Specificity for Device Subgroup Analysis** The forest plot presents the pooled specificity estimates of smartwatches for detecting atrial fibrillation, stratified by device brand. The pooled specificity across the included studies was 96.40% (95% CI: 94.12% to 97.82%).

Our analysis of smartwatch specificity revealed a pooled specificity of 96.12% (95% CI: 94.12%-97.82%; I = 99%) for detecting AF. The subgroup analysis based on smartwatch type showed that the Apple Watch had a specificity of 96.97% (95% CI: 93.20%-98.68%; I = 87%), Samsung 95.81% (95% CI: 91.31%-98.03%; I = 93%), Withing’s 95.03% (95% CI: 84.26%-98.56%; I = 93%), Fitbit 79.29% (95% CI: 71.62%-85.67%), Scan Watch 100% (95% CI: 96.67%-100%), Seiko Epson Smartwatch 90.64% (95% CI: 86.17%-94.04%), Amazfit 98.87% (95% CI: 94.64%-99.77%; I = 99%), and Garmin Forerunner 945 88.24% (95% CI: 78.13%-94.68%) (Figure 4). Individual smartwatch diagnostic results are summarized in the Central Illustration.

**Figure 7:**
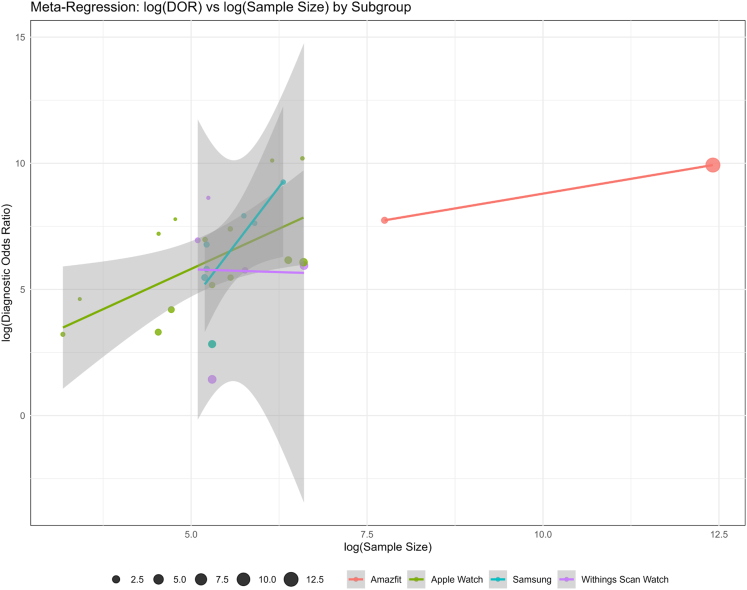
**Meta-Regression: log (DOR) vs log (Sample Size) by Device** A positive association was observed in several subgroups. Apple Watch and Amazfit studies showed higher diagnostic ORs in larger samples, suggesting sample size as a driver of observed heterogeneity. DOR = diagnostic OR.

**Figure 5:**
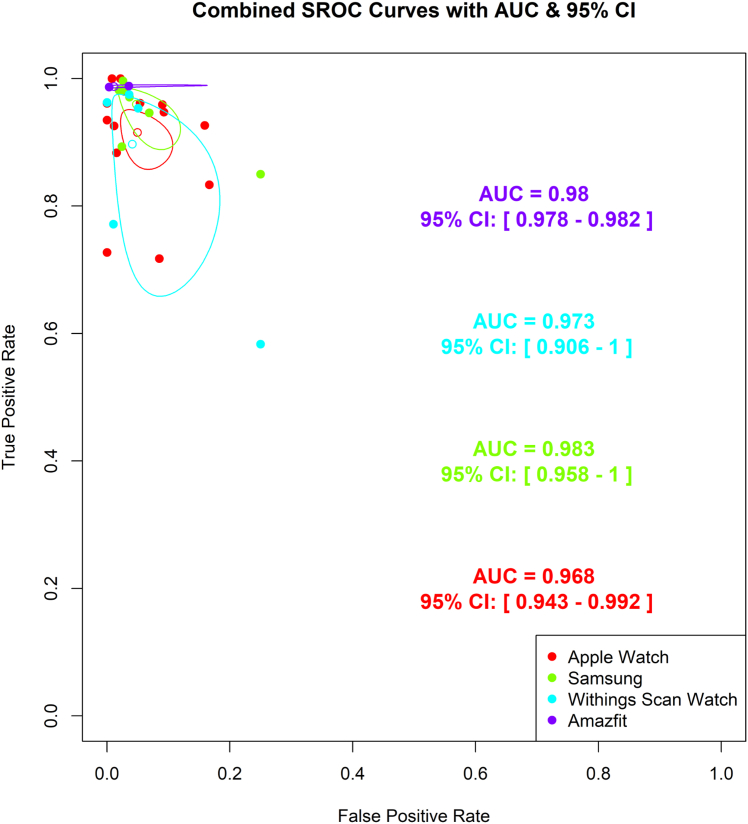
**SROC Curves for Smartwatch Subgroups** Each curve represents the diagnostic performance of a specific brand: Apple Watch (AUC: 0.968; SD: 0.01), Samsung (AUC: 0.983; SD: 0.013), Withing’s (AUC: 0.967; SD: 0.05), and Amazfit (AUC: 0.98; SD: 0.001). The plot highlights excellent overall diagnostic accuracy across all devices. SROC = summary receiver-operating characteristic.

**Figure 6:**
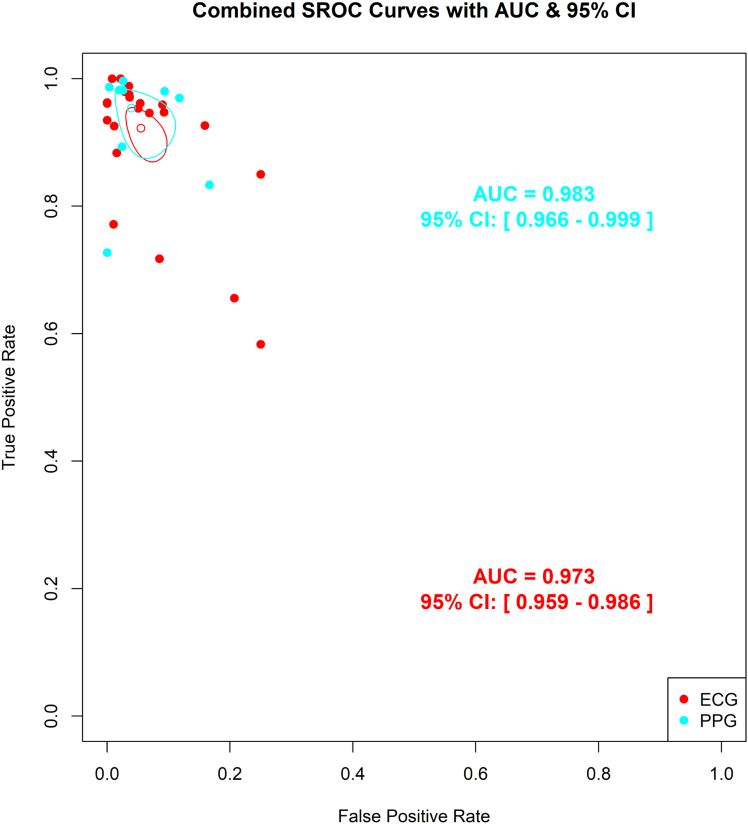
**Pooled SROC Curves by Smartwatch Detection Technology** This figure illustrates the pooled diagnostic performance of smartwatches using different detection technologies—ECG and PPG—for the detection of AF. Both indicate high diagnostic accuracy for both modalities. AF = atrial fibrillation; ECG = electrocardiogram; PPG = photoplethysmography; other abbreviation as in Figure 5.

**Central Illustration:**
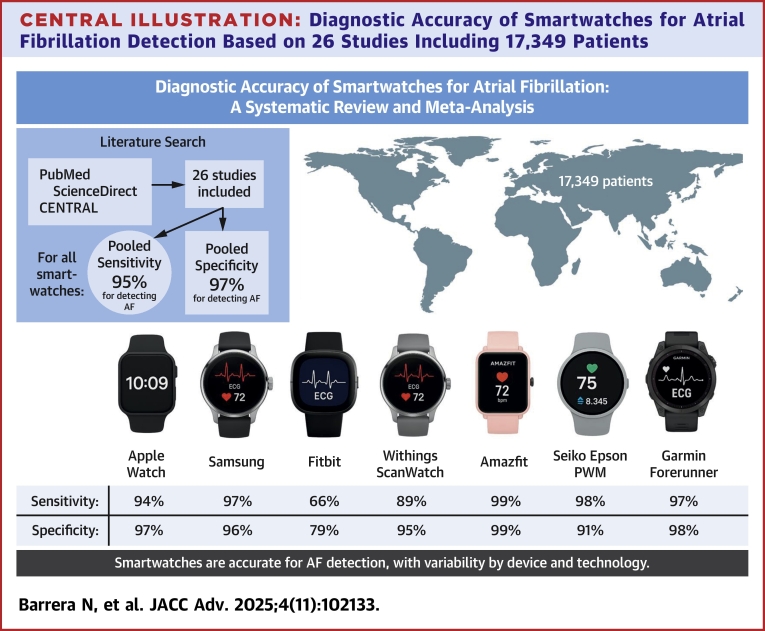
Diagnostic Accuracy of Smartwatches for Atrial Fibrillation Detection Based on 26 Studies Including 17,349 Patients Showing excellent pooled sensitivity and specificity values by technology. Abbreviation as in Figure 6.

### Negative predictive value

Our analysis revealed a pooled NPV of 97.56% (95% CI: 95.59%-98.66%) for smartwatches in detecting atrial AF, with significant heterogeneity observed (I^2^ = 99%) (Supplemental Figure 7).

### Positive predictive value

Regarding the PPV, the analysis revealed a PPV of 92.51% (95% CI: 88.03%-95.40%), also exhibiting significant heterogeneity (I^2^ = 96%) (Supplemental Figure 8).

### SROC curves

We generated 2 SROCs for our analysis. The first SROC summarized the accuracy of different smartwatches, while the second compared the 2 baseline technologies used in the smartwatches: PPG and ECG. According to the SROC curves, the Samsung smartwatch demonstrated the highest diagnostic accuracy with an AUC of 0.98 (95% CI: 0.97%-0.99%), followed closely by Amazfit AUC 0.98 (95% CI: 0.978%-0.982%), Apple Watch AUC 0.97 (95% CI: 0.94%-0.99%), and Withing’s AUC 0.97 (95% CI: 0.91%-1.0%) (Figure 5). When comparing the technologies, PPG-based smartwatches showed similar diagnostic accuracy, AUC 0.98 (95% CI: 0.97%-0.99%) to ECG-based technologies AUC 0.97 (95% CI: 0.96%-0.98%) (Figure 6). Smartwatches included in only one study were excluded from the SROC curves.

### Diagnostic odds ratio and meta-regression

The pooled DORs was 386, 59 (95% CI: 101,33%-1474%; I^2^: 98%), showing an overall strong diagnostic performance with high heterogeneity. Individual study DORs varied widely from as low as 4.20 to as high as 24576. CIs were broad, reflecting variability in sample size and test methodology (Supplemental Figure 9).

Using Amazfit as the reference subgroup, our meta-regression found that Amazfit had significantly higher odds of diagnosing AF than the baseline; the odds of diagnostic accuracy of the Samsung smartwatch were not significantly different from that of Amazfit. However, both Withings and Apple Watch demonstrated significantly lower diagnostic accuracy than Amazfit (Supplemental Figure 10, Supplemental Table 2). After adjusting for log-transformed sample size, no significant differences in diagnostic accuracy were observed between smartwatch brands compared to the reference group (Amazfit). Sample size was significantly associated with diagnostic accuracy (β = 0.92; *P* = 0.022). Inclusion of sample size in the model reduced heterogeneity, and subgroup effects were no longer statistically significant (Figure 7, Supplemental Table 3).

Meta-regression of the baseline technology, using ECG as the reference subgroup, our model found that ECG-based devices had similar odds of diagnosing AF compared to the baseline.(Supplemental Figure 11, Supplemental Table 3).

### Sub-group analysis by device generation

A subgroup analysis of the sensitivity revealed a significant reduction in heterogeneity, particularly for the Apple Watch smartwatch. The observed heterogeneity across the studies that analyzed the Apple Watch combined with the Kardia Band and Apple Watch 6 was I^2^ = 0%. Apart from these 2 subgroups, heterogeneity was still observed in the other subgroups (Supplemental Figures 11 and 12).

### Utility of smartwatches in the secondary setting

In an exploratory outcome, we also examined the utility of smartwatches for detecting AF as a secondary prevention method following a stroke event. We identified only one study investigating the Pulse Watch, which reported an accuracy of 92.9% (95% CI: 85.3% to 97.4%) for AF detection, with a sensitivity of 60% (95% CI: 14.7% to 94.7%) and a specificity of 95% (95% CI: 87.7% to 98.6%) in detecting AF after stroke or transient ischemic attacks.^32^

## Discussion

Our review found that smartwatches exhibit high accuracy, sensitivity, and specificity in detecting AF. Among the various models assessed, the Samsung smartwatch demonstrated the highest diagnostic accuracy, followed by Amazfit, Apple Watch, and Withings. Notably, PPG-based smartwatches and ECG-based models had a similar diagnostic accuracy. While multiple brands have shown high performance, the Apple Watch remains the most extensively studied device, with the largest body of clinical evidence supporting its use in both research and real-world settings.

A primary challenge in modern cardiology is increasing public awareness of the risks associated with disease development, which can facilitate early detection and preventive measures. In the case of AF, patients often have a low understanding of its symptoms and health implications. This lack of awareness has been linked to poorer prognoses, with evidence suggesting that it may lead to worse outcomes.^37^^,^^38^ To increase AF detection, ambulatory devices like Holter monitors are utilized in high-risk populations; however, their effectiveness is constrained by the limited duration of monitoring. In contrast, newer technologies such as insertable cardiac monitors have demonstrated superior ability in detecting AF in patients with cryptogenic stroke.^39^

The integration of rhythm monitors into wearable devices aims to enhance the utilization of rhythm monitoring within the general population. Current wearable technologies, including smartwatches and smartphones, have become widely adopted.^40^ However, despite the increasing use of these technologies for detecting AF, concerns about false positives persist, potentially surpassing systematic findings. It is important to highlight that the threshold of subclinical AF necessary for detection by smartwatches remains unclear. Additionally, the extent to which brief episodes of subclinical AF contribute to stroke risk is uncertain, and the specific duration of subclinical AF that would justify anticoagulant therapy has not been definitively established.^41^^,^^42^ Therefore, comprehensive research is needed to determine whether smartwatch-detected subclinical AF or low-burden episodes correlate with an increased risk of stroke.^43^^,^^44^

Our analysis indicated high diagnostic accuracy across all smartwatches, with most models — such as Samsung, Apple Watch, Scan Watch, and Amazfit — reporting sensitivities above 90%. A previous review by Shahid et al similarly found high sensitivity (95%) and specificity (95%) for the Apple Watch in detecting AF.^8^ Another review by Elbey et al in 2021 concluded that smartwatch technology is noninferior to 12-lead ECGs, Holter monitors, or patch ECGs in detecting AF.^45^ Shahid et al suggested a linear relationship between the technology series of the Apple Watch and its diagnostic accuracy, with newer models demonstrating improved performance.^8^ Similarly, our subgroup analysis by device generation indicated a moderate decrease in heterogeneity. This suggests that advancements in device software across different generations of smartwatches may play a role in achieving more consistent diagnostic performance.

As a complementary analysis, a DOR was conducted to provide a single performance indicator by combining sensitivity and specificity. DOR is particularly useful in the context of heterogeneous studies, as it summarizes the odds of a positive test result in individuals with AF compared to those without, regardless of disease prevalence. Our DOR results show that the smartwatch has excellent overall discriminatory power to detect AF.

Additionally, our meta-regression analysis identified study sample size as a significant factor influencing diagnostic accuracy, while variations in device brand or baseline technology were not statistically significant. These results imply that study-level factors, particularly sample size, play a crucial role in the observed heterogeneity, rather than intrinsic differences between devices. Larger studies often report more favorable diagnostic performance, likely due to enhanced methodological rigor, greater statistical precision, and more reliable estimates across a broader range of patient populations, in contrast to smaller studies. These findings highlight the importance of study-level factors in shaping the diagnostic performance and support the need for large, prospective, and uniform studies in future smartwatch research.

Despite these promising results, users should remain aware of the current limitations associated with smartwatch use for AF detection. Some studies excluded patients from analysis due to poor recordings, suggesting that errors may still occur with continued use.^46^ Additionally, the prevalence of AF in selected study populations is often higher than in the general population, which may result in higher PPV and lower NPV for smartwatches in broader contexts. As demonstrated by Cheung et al, this discrepancy indicates that a significant proportion of smartwatch users may undergo unnecessary testing and potentially unwarranted therapies.^46^ As smartwatches become increasingly integrated into everyday consumer health, it is essential to understand their usability, cost-effectiveness, and potential psychological effects to effectively incorporate them into clinical practice. From a usability standpoint, while many users feel comfortable navigating smartwatch interfaces, older patients and individuals with low digital literacy may struggle to interpret AF notifications. This can create psychological distress, as false alarms or ambiguous alerts may induce anxiety and lead to unnecessary medical attention. Additionally, the substantial volume of data generated by smartwatches could increase the workload for an already strained clinician workforce, potentially resulting in unnecessary medical referrals, medical appointments, and premature initiation of oral anticoagulation.^43^^,^^47^ In contrast, younger patients who typically have a low pretest probability and are the most frequent users of smartwatches may be disproportionately affected by false positives. The economic viability of utilizing smartwatches for the detection of AF in high-risk populations remains a topic of ongoing debate. The U.S. Preventive Services Task Force does not support widespread screening for AF.^43^ However, recent research employing a microsimulation decision-analytic model indicates that screening with wearable devices may prove to be more cost-effective than both abstaining from screening altogether and employing traditional AF screening methods. This emerging evidence suggests a potential reevaluation of current screening recommendations in light of advancements in wearable technology.^48^ We look forward to the results of the REACT-AF trial (NCT05836987) that compares continuous oral anticoagulation vs 30-day oral anticoagulation after an AF episode detected by a smartwatch, and also the HEARTLINE-A study (NCT04276441) that evaluated if early detection of AF with an Apple Watch reduces the risk of thromboembolic events in the real-world setting.

### Study Limitations

The current study utilized a robust methodological approach to evaluate the effectiveness of smartwatch technology in detecting AF. Nonetheless, several limitations warrant consideration. First, although a substantial degree of heterogeneity was observed across studies, our subgroup and meta-regression analyses identified device generation and sample size as key contributors. Specifically, larger sample sizes were associated with higher diagnostic accuracy, suggesting that study-level factors significantly influenced effect estimates. Second, the different prevalence rates of AF among the samples may have resulted in significant variability in the efficacy of the smartwatches. Third, there was a limited number of studies investigating the use of certain smartwatches, which led to their exclusion from the SROC curves and meta-regression analysis. Finally, only one study examined the use of smartwatches for detecting AF as a secondary prevention strategy, restricting our ability to draw meaningful conclusions regarding their application in this context.

## Conclusions

Our study demonstrated that smartwatch technology exhibits high diagnostic accuracy for detecting AF. Our analysis indicated that PPG-based smartwatches exhibited comparable diagnostic accuracy to ECG-based smartwatches. However, meta-regression analysis revealed that study sample size significantly contributed to diagnostic performance, with larger studies reporting higher accuracy. Further research is necessary to evaluate the efficacy of smartwatches in real-world, low-prevalence settings, where predictive value may vary. Additionally, advancements in smartwatch technology are essential to enhance their performance and minimize the impact of artifacts on diagnostic accuracy.

Perspectives **COMPETENCY IN PATIENT CARE AND PROCEDURAL SKILLS:** Wearable devices such as smartwatches are emerging as accessible tools for AF detection. Our findings show that these technologies, particularly those using ECG and PPG sensors, can identify AF with high sensitivity and specificity. For clinicians, this means that smartwatches may serve as a practical option for rhythm screening. Still, careful interpretation is necessary to avoid unnecessary testing triggered by false positives, particularly in populations with low baseline prevalence.

**TRANSLATIONAL OUTLOOK:** As digital health tools gain traction, smartwatches offer a scalable solution for AF screening. This meta-analysis supports their use in targeted screening programs. However, broader implementation requires more data—particularly on how these tools affect health care utilization, long-term outcomes, and patient well-being. Future research should also consider cost-effectiveness and patient engagement to ensure that integration of wearable technologies into clinical workflows is both beneficial and sustainable.

## Funding support and author disclosures

The authors have reported that they have no relationships relevant to the contents of this paper to disclose.
