# Supplementary material for: Accuracy of Smartwatches in the Detection of Atrial Fibrillation: A Systematic Review and Diagnostic Meta-Analysis
Source: JACC Adv. 2025 Nov 3;4(11):102133. doi: 10.1016/j.jacadv.2025.102133 (PMC12713314; doi:10.1016/j.jacadv.2025.102133)
Supplement: Supplemental Material [file mmc1.docx]

**Prisma-DTA Checklist Item**

| **Section/topic** | **#** | **PRISMA-DTA Checklist Item** | **Reported on page #** |
| --- | --- | --- | --- |
| **TITLE / ABSTRACT** | | |  |
| Title | 1 | Identify the report as a systematic review (+/- meta-analysis) of diagnostic test accuracy (DTA) studies. | 1 |
| Abstract | 2 | Abstract: See PRISMA-DTA for abstracts. | 2 |
| **INTRODUCTION** | | |  |
| Rationale | 3 | Describe the rationale for the review in the context of what is already known. | 6,7 |
| Clinical role of index test | D1 | State the scientific and clinical background, including the intended use and clinical role of the index test, and if applicable, the rationale for minimally acceptable test accuracy (or minimum difference in accuracy for comparative design). | 6,7 |
| Objectives | 4 | Provide an explicit statement of question(s) being addressed in terms of participants, index test(s), and target condition(s). | 6,7 |
| **METHODS** | | |  |
| Protocol and registration | 5 | Indicate if a review protocol exists, if and where it can be accessed (e.g., Web address), and, if available, provide registration information including registration number. | 8 |
| Eligibility criteria | 6 | Specify study characteristics (participants, setting, index test(s), reference standard(s), target condition(s), and study design) and report characteristics (e.g., years considered, language, publication status) used as criteria for eligibility, giving rationale. | 8,9 |
| Information sources | 7 | Describe all information sources (e.g., databases with dates of coverage, contact with study authors to identify additional studies) in the search and date last searched. | 9 |
| Search | 8 | Present full search strategies for all electronic databases and other sources searched, including any limits used, such that they could be repeated. | N/A |
| Study selection | 9 | State the process for selecting studies (i.e., screening, eligibility, included in systematic review, and, if applicable, included in the meta-analysis). | 8,9 |
| Data collection process | 10 | Describe method of data extraction from reports (e.g., piloted forms, independently, in duplicate) and any processes for obtaining and confirming data from investigators. | 8,9 |
| Definitions for data extraction | 11 | Provide definitions used in data extraction and classifications of target condition(s), index test(s), reference standard(s) and other characteristics (e.g. study design, clinical setting). | 9 |
| Risk of bias and applicability | 12 | Describe methods used for assessing risk of bias in individual studies and concerns regarding the applicability to the review question. | 9,10 |
| Diagnostic accuracy measures | 13 | State the principal diagnostic accuracy measure(s) reported (e.g. sensitivity, specificity) and state the unit of assessment (e.g. per-patient, per-lesion). | 10,11 |
| Synthesis of results | 14 | Describe methods of handling data, combining results of studies and describing variability between studies. This could include, but is not limited to: a) handling of multiple definitions of target condition. b) handling of multiple thresholds of test positivity, c) handling multiple index test readers, d) handling of indeterminate test results, e) grouping and comparing tests, f) handling of different reference standards | 10, 11 |

| **Section/topic** | **#** | **PRISMA-DTA Checklist Item** | **Reported on page #** |
| --- | --- | --- | --- |
| Meta-analysis | D2 | Report the statistical methods used for meta-analyses, if performed. | 10,11 |
| Additional analyses | 16 | Describe methods of additional analyses (e.g., sensitivity or subgroup analyses, meta-regression), if done, indicating which were pre-specified. | 10,11 |
| **RESULTS** | | |  |
| Study selection | 17 | Provide numbers of studies screened, assessed for eligibility, included in the review (and included in meta-analysis, if applicable) with reasons for exclusions at each stage, ideally with a flow diagram. | 11 |
| Study characteristics | 18 | For each included study provide citations and present key characteristics including: a) participant characteristics (presentation, prior testing), b) clinical setting, c) study design, d) target condition definition, e) index test, f) reference standard, g) sample size, h) funding sources | 12, Figure 1 |
| Risk of bias and applicability | 19 | Present evaluation of risk of bias and concerns regarding applicability for each study. | 12 |
| Results of individual studies | 20 | For each analysis in each study (e.g. unique combination of index test, reference standard, and positivity threshold) report 2x2 data (TP, FP, FN, TN) with estimates of diagnostic accuracy and confidence intervals, ideally with a forest or receiver operator characteristic (ROC) plot. | 13,14 |
| Synthesis of results | 21 | Describe test accuracy, including variability; if meta-analysis was done, include results and confidence intervals. | 13,14 |
| Additional analysis | 23 | Give results of additional analyses, if done (e.g., sensitivity or subgroup analyses, meta-regression; analysis of index test: failure rates, proportion of inconclusive results, adverse events). | 15,16 |
| **DISCUSSION** | | |  |
| Summary of evidence | 24 | Summarize the main findings including the strength of evidence. | 17 |
| Limitations | 25 | Discuss limitations from included studies (e.g. risk of bias and concerns regarding applicability) and from the review process (e.g. incomplete retrieval of identified research). | 21 |
| Conclusions | 26 | Provide a general interpretation of the results in the context of other evidence. Discuss implications for future research and clinical practice (e.g. the intended use and clinical role of the index test). | 17-20 |
| **FUNDING** | | |  |
| Funding | 27 | For the systematic review, describe the sources of funding and other support and the role of the funders. | 1 |

*Adapted From:*  McInnes MDF, Moher D, Thombs BD, McGrath TA, Bossuyt PM, The PRISMA-DTA Group (2018). Preferred Reporting Items for a Systematic Review and Meta-analysis of Diagnostic Test Accuracy Studies: The PRISMA-DTA Statement. JAMA. 2018 Jan 23;319(4):388-396. doi: 10.1001/jama.2017.19163.

**Supplemental Figure 1. Publication bias funnel plot**

**
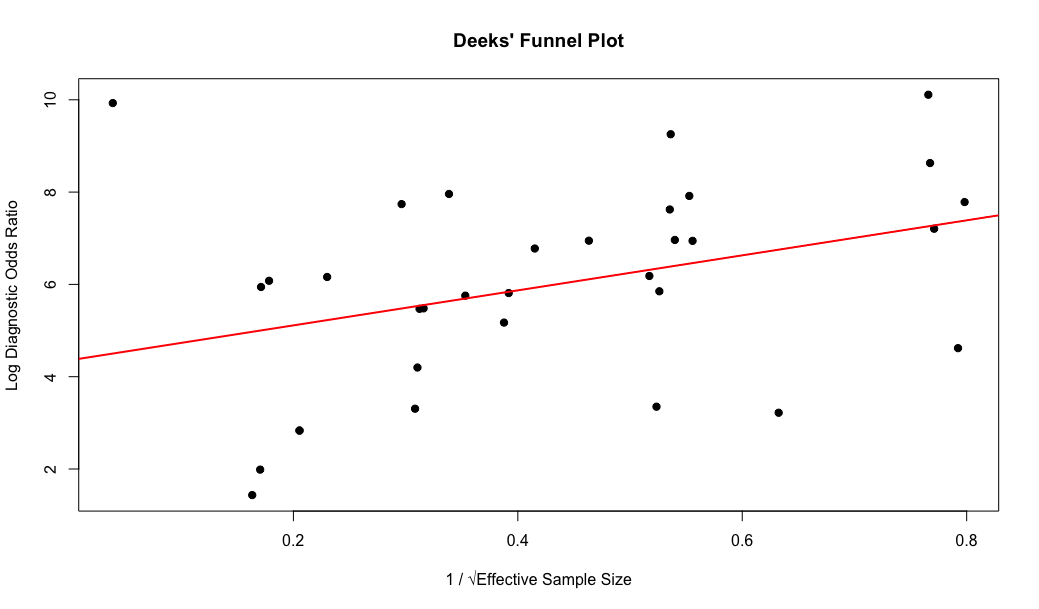
**

Deeks’ funnel plot assessing publication bias among the included diagnostic accuracy studies. Each point represents a study, plotting the log diagnostic odds ratio against the inverse square root of the effective sample size. The red regression line suggests a mild asymmetry, with a statistically significant slope (p = 0.048), indicating possible publication bias.

**Supplemental Table 1. Detailed evaluation of publication bias with Deek’s Test**

| tau^2 (estimated amount of residual heterogeneity): 4.0201 (SE = 1.2096)  tau (square root of estimated tau^2 value): 2.0050  I^2^ (residual heterogeneity / unaccounted variability): 92.86%  H^2^ (unaccounted variability / sampling variability): 14.01  R^2^ (amount of heterogeneity accounted for): 7.78% |
| --- |
| Test for Residual Heterogeneity:  QE(df = 31) = 1284.8911, p-val < .0001 |
| Test of Moderators (coefficient 2):  QM(df = 1) = 3.8905, p-val = 0.0486 |

Evaluation shows that the potential publication bias due to effect size accounting for (7.78%) of the perceived heterogeneity.

**Supplemental Figure 2**. Leave-one-out analysis for sensitivity


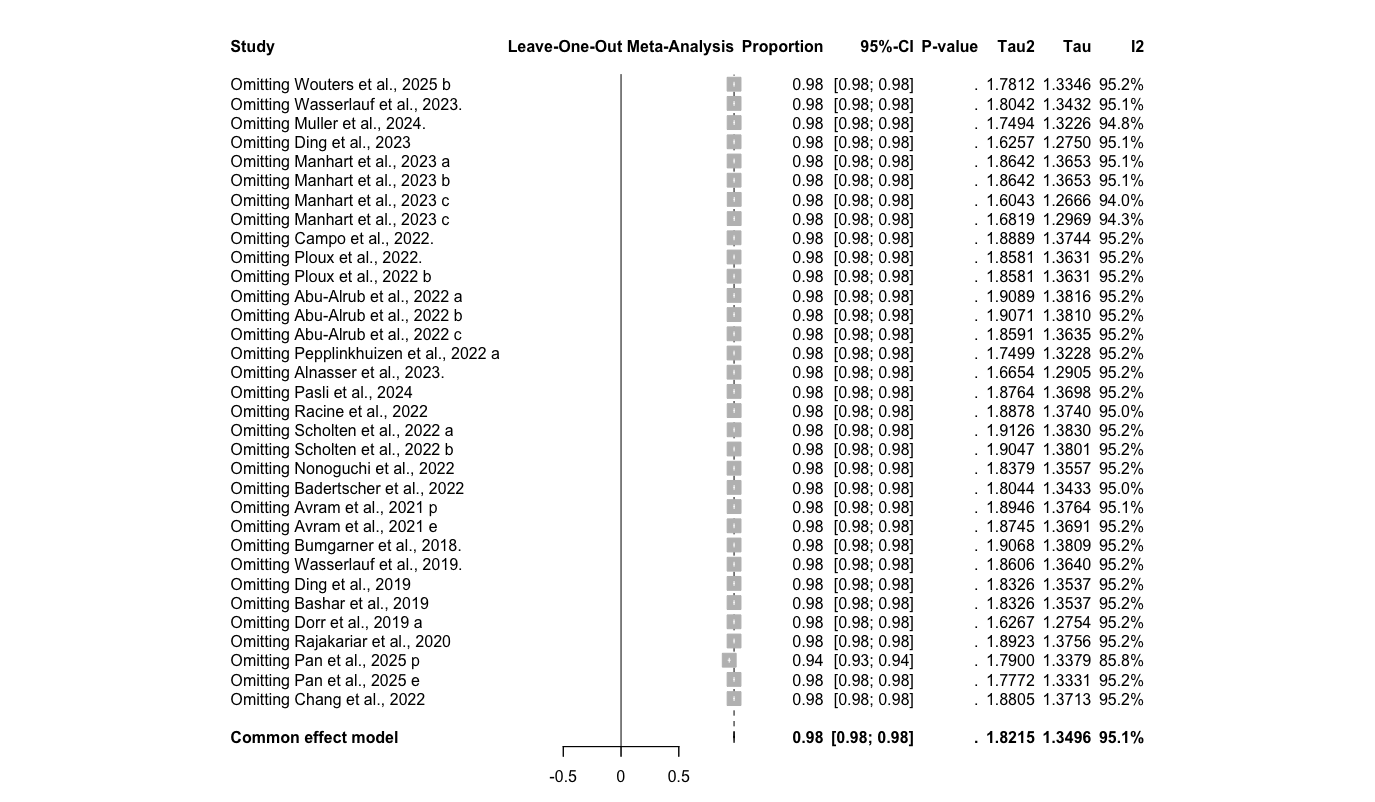


**Supplemental Figure 3**. Leave-one-out analysis for specificity


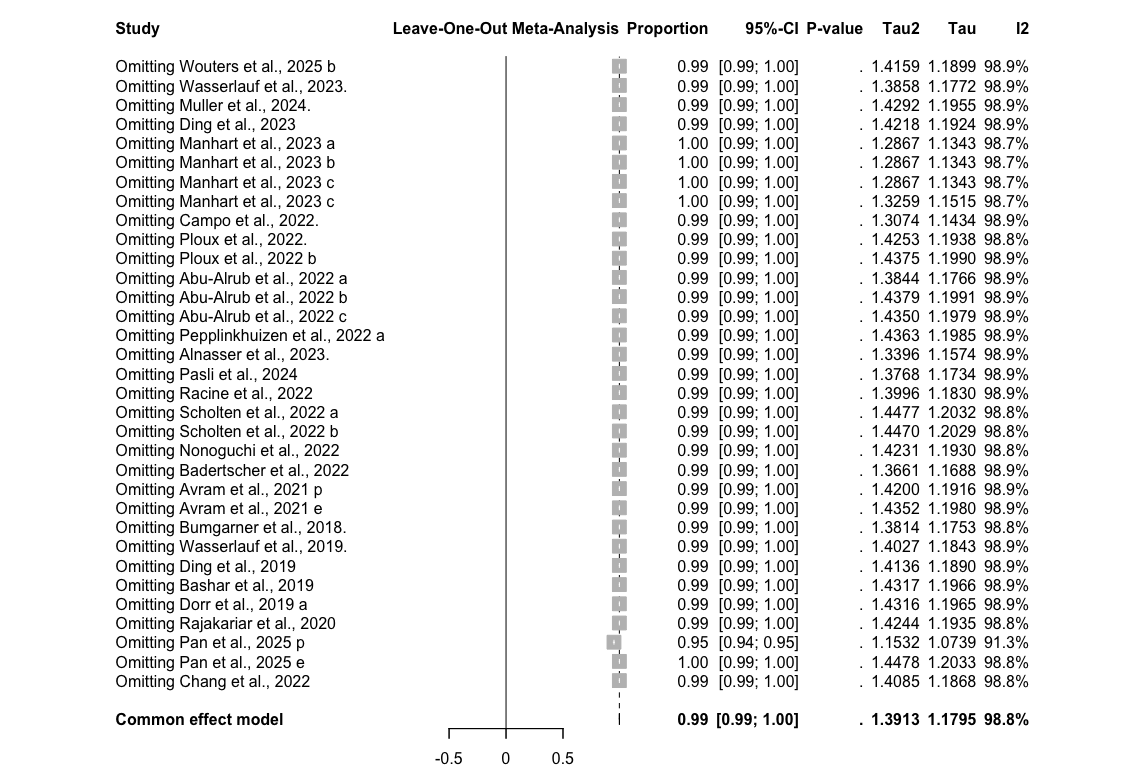


**Supplemental Figure 4. Leave-one-out analysis for positive predictive value**


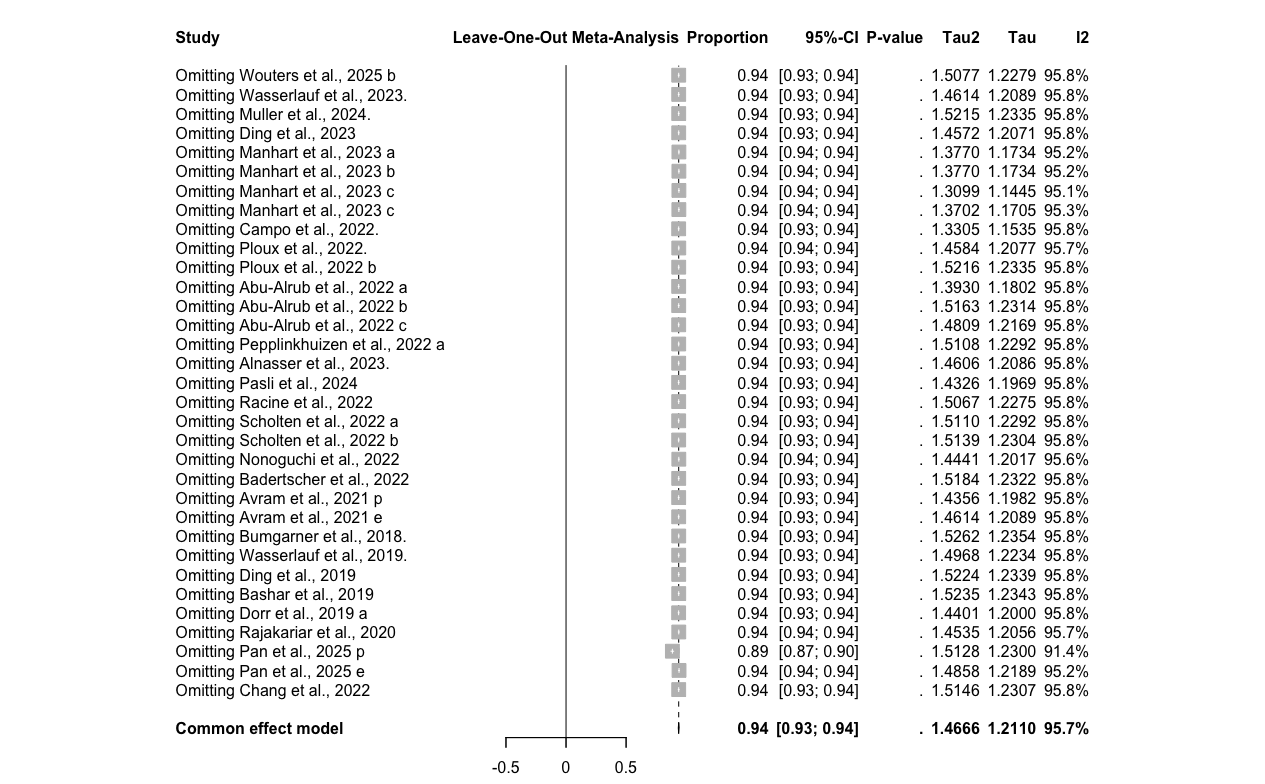


**Supplemental Figure 5. Leave-one-out analysis for negative predictive value**

**
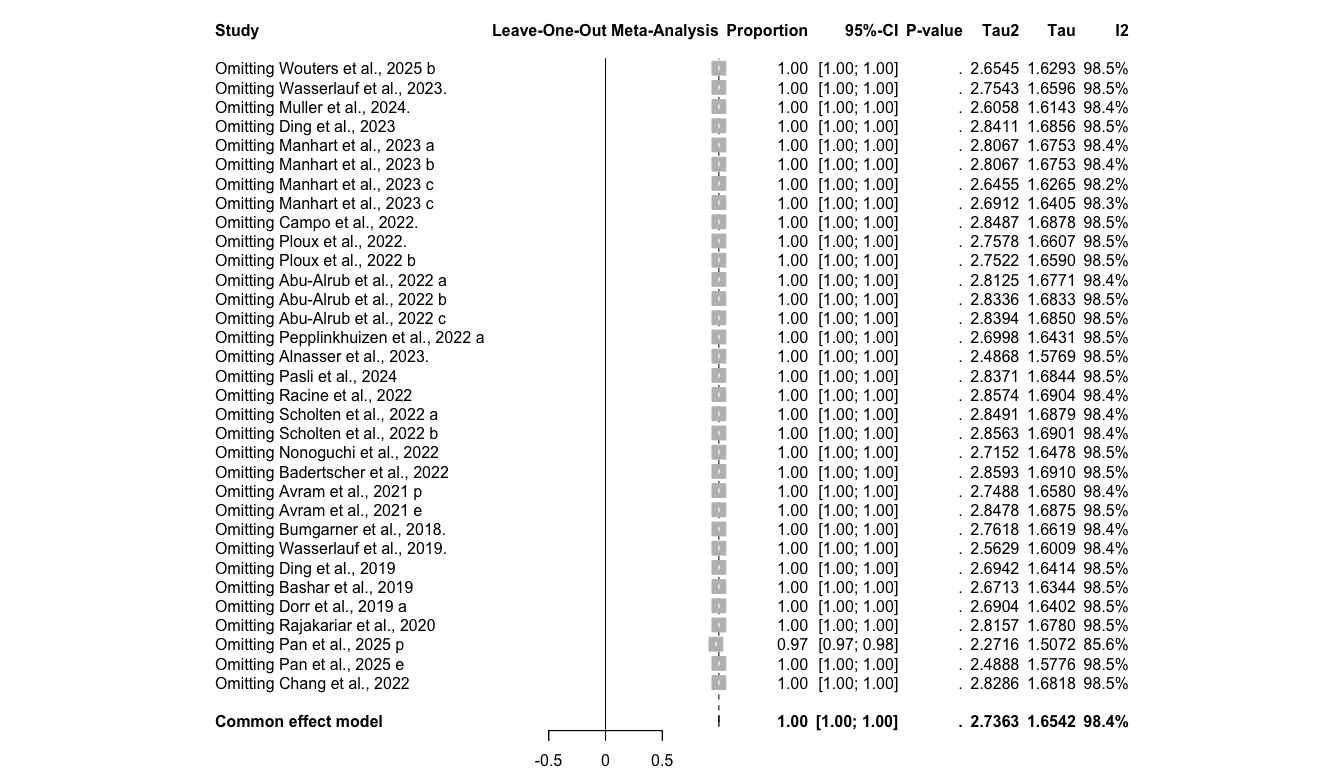
**

**Supplemental Figure 6. The GRADE (Good Research for Comparative Effectiveness) framework**

**
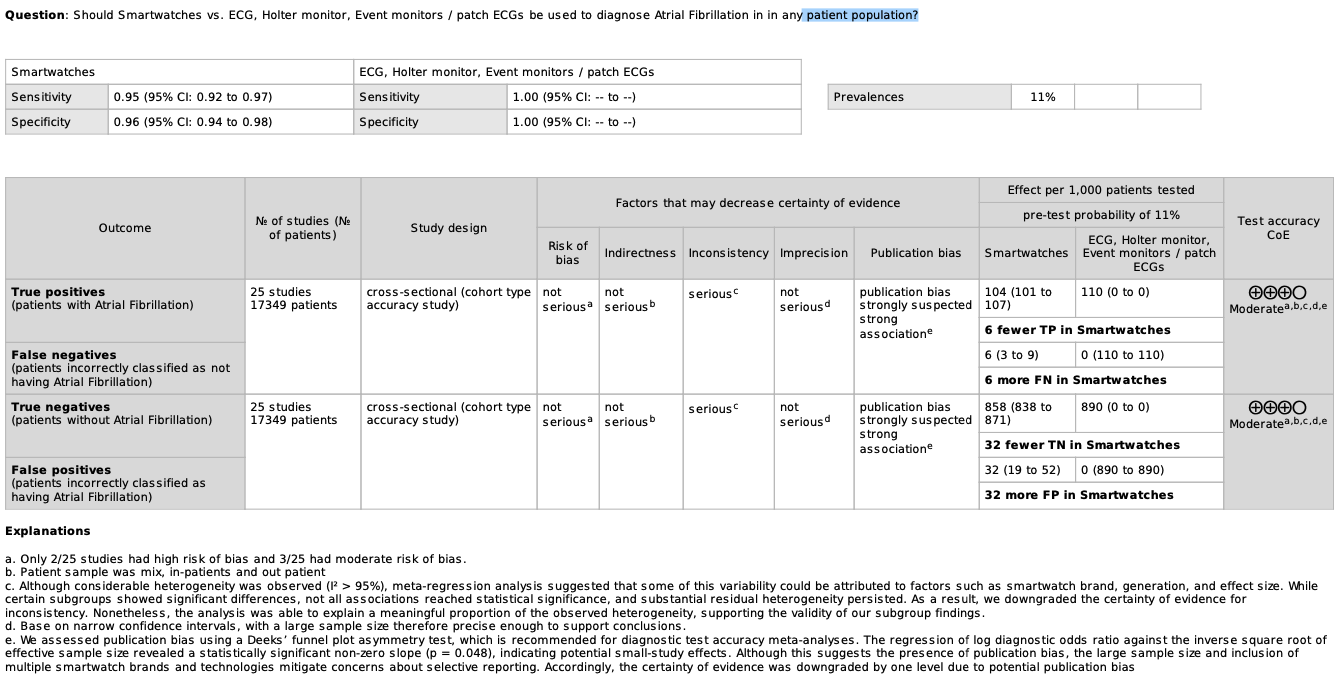
**

**Supplemental Figure 7. Forest Plot of Negative Predictive Value for Smartwatch Detection of Atrial Fibrillation**


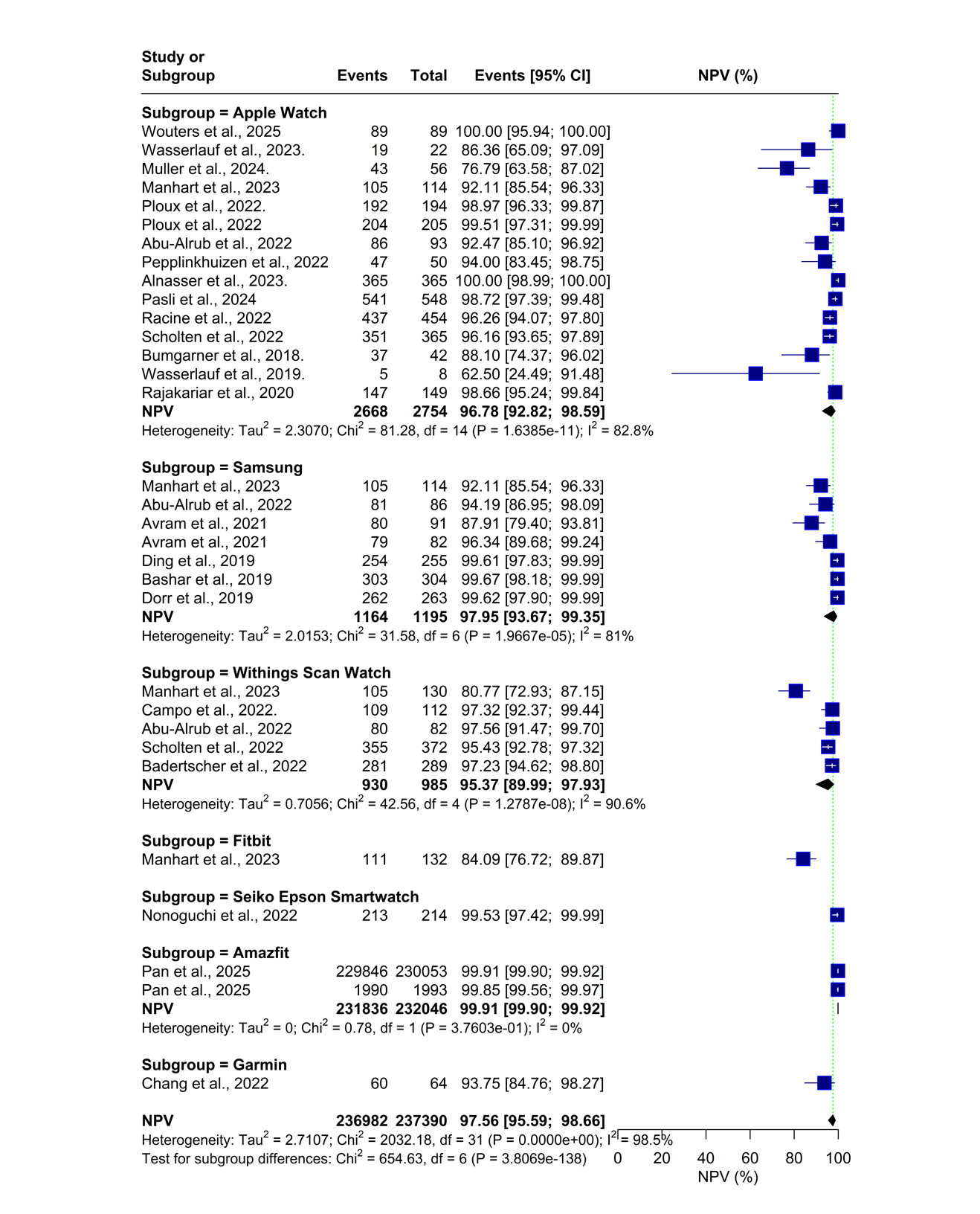


**Supplemental 8. Forest Plot of Positive Predictive Value for Smartwatch Detection of Atrial Fibrillation**


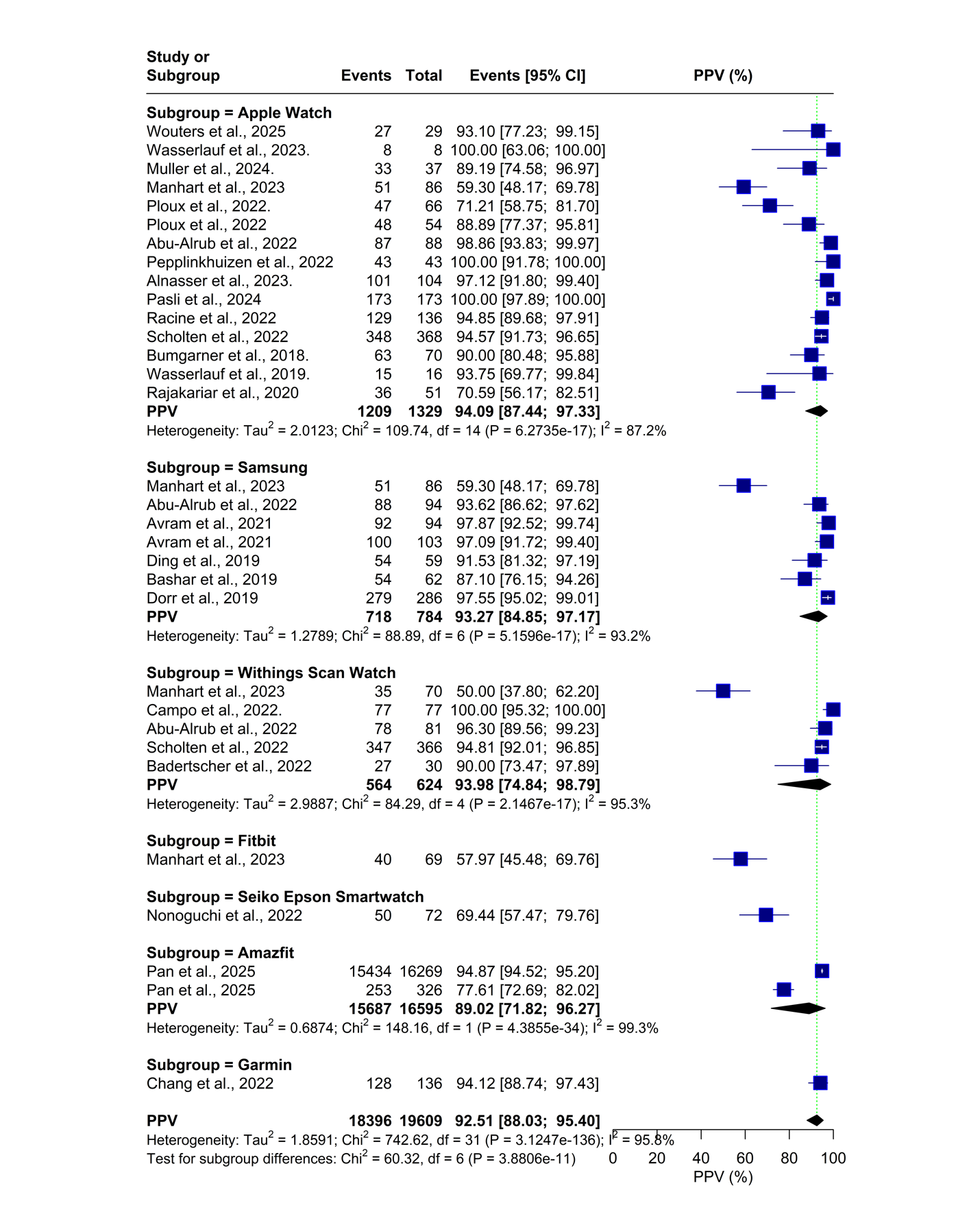


**Supplemental Figure 9. Diagnostic Accuracy of Smartwatches for AF Detection: Pooled DORs Across Studies**

**
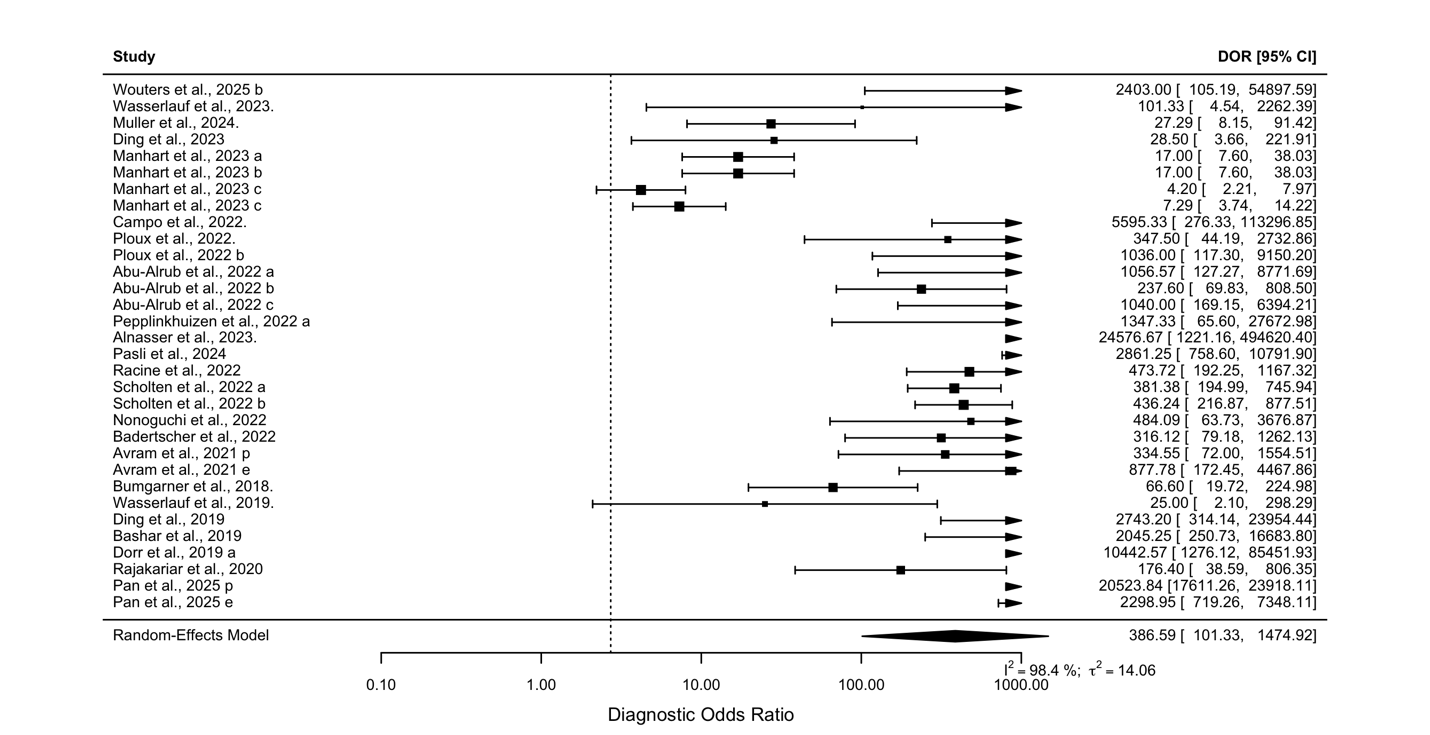
**

**Supplemental Figure 10. Meta-Regression Plot Showing the Impact of Smartwatch Brand on Diagnostic Accuracy**


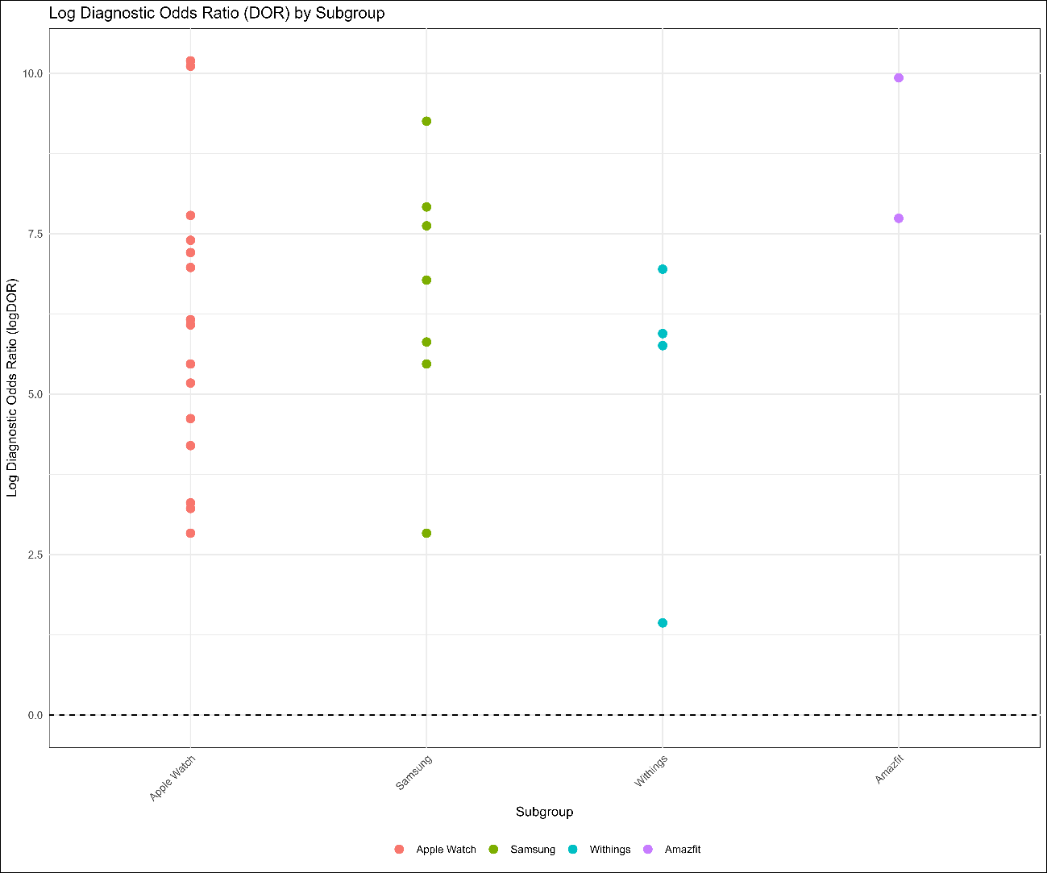


**Supplemental Table 2. Meta-Regression Results Assessing the Impact of Smartwatch Brand on Diagnostic Accuracy**.

|  | estimate | se | tval | df | pval | ci.lb | ci.ub |
| --- | --- | --- | --- | --- | --- | --- | --- |
| intercept | 8.8807 | 1.4385 | 6.1735 | 24 | <.0001 | 5.9117 | 11.8496 *** |
| Subgroup Apple Watch | -3.0465 | 1.5509 | -1.9643 | 24 | 0.0612 | -6.2474 | 0.1544 ‘.’ |
| Subgroup Samsung | 2.5042 | 1.6559 | -1.5123 | 24 | 0.1435 | -5.9217 | 0.9134 |
| Subgroup Withings | -3.9586 | 1.7762 | -2.2287 | 24 | 0.0355 | -7.624 | -0.2927 * |

Significance. codes: 0 ‘***’ 0.001 ‘**’ 0.01 ‘*’ 0.05 ‘.’ 0.1 ‘ ’ 1

**Supplemental Table 3. Meta-Regression of Dors Assessing the Impact of the Study’s Sample Size on the Diagnostic Accuracy of Smartwatches**

| Variable | Estimate | Std. Error | T-value | Df | P-value | 95% ci (lower) | 95% ci (upper) |
| --- | --- | --- | --- | --- | --- | --- | --- |
| Intercept (Reference) | -0.5124 | 4.0712 | -0.1259 | 24 | 0.9009 | -8.9149 | 7.8902 |
| Subgroup: Apple Watch | 1.4803 | 2.3501 | 0.6299 | 24 | 0.5347 | -3.3701 | 6.3307 |
| Subgroup: Samsung | 1.7714 | 2.3301 | 0.7602 | 24 | 0.4545 | -3.0377 | 6.5806 |
| Subgroup: Withings Scan Watch | 0.7319 | 2.3411 | 0.3126 | 24 | 0.7573 | -4.0998 | 5.5636 |
| log(Sample Size) | 0.9220 | 0.3778 | 2.4408 | 24 | 0.0224 | 0.1424 | 1.7017 |

The meta regression model results indicate the impact of the type of smartwatch, adjusted for the sample size, on the overall diagnostic accuracy.

**Supplemental Figure 11. Log Diagnostic Odds Ratio by Baseline Technology (ECG vs. PPG)**


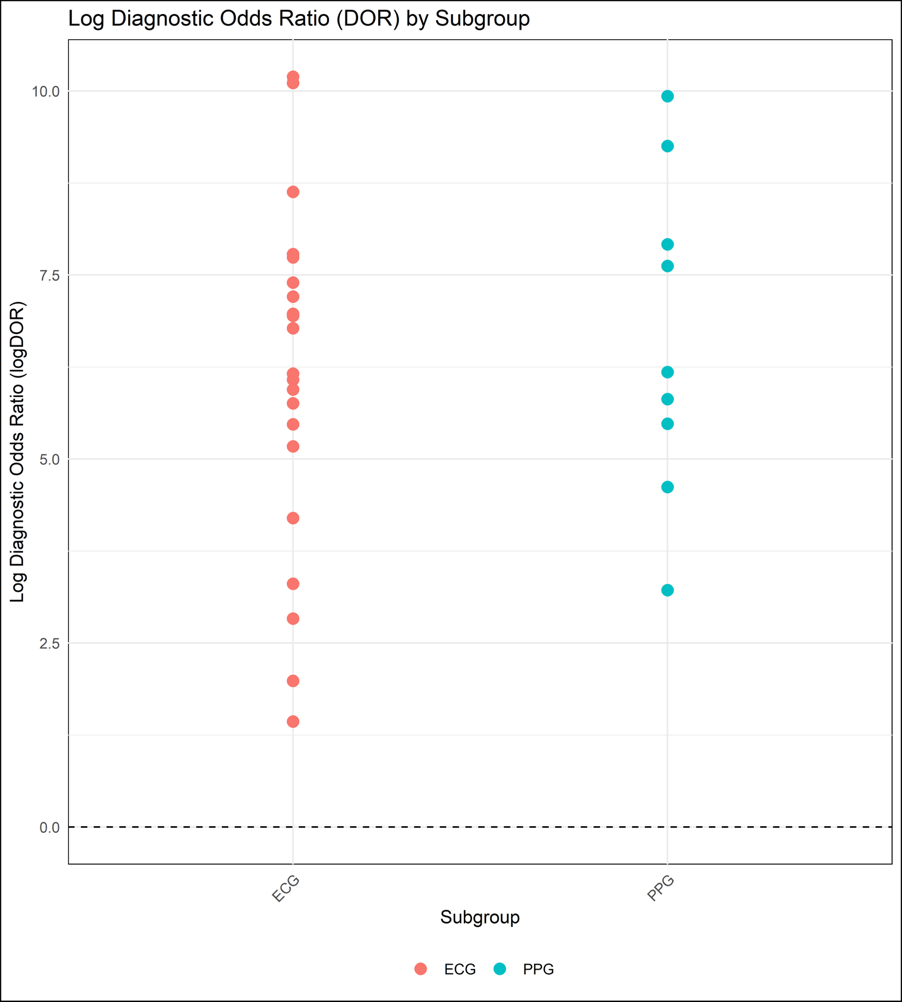


**Supplemental Table 3**. **Meta-Regression Results Assessing the Impact of Baseline Detection Technology (PPG vs. ECG) on Smartwatch Diagnostic Accuracy**

|  | estimate | se | tval | df | pval | ci.lb | ci.ub |
| --- | --- | --- | --- | --- | --- | --- | --- |
| intercept | 5.6851 | 0.4782 | 11.8876 | 29 | <.0001 | 4.7070 | 6.6632 *** |
| Subgroup PPG | 1.3022 | 0.9635 | 1.3514 | 29 | 0.1870 | -0.6685 | 3.2728 |

Significance codes: 0 ‘***’ 0.001 ‘**’ 0.01 ‘*’ 0.05 ‘.’ 0.1 ‘ ’ 1

**Supplemental Figure 12. Forest Plot of the Pooled Sensitivity Stratified by Device Generation**


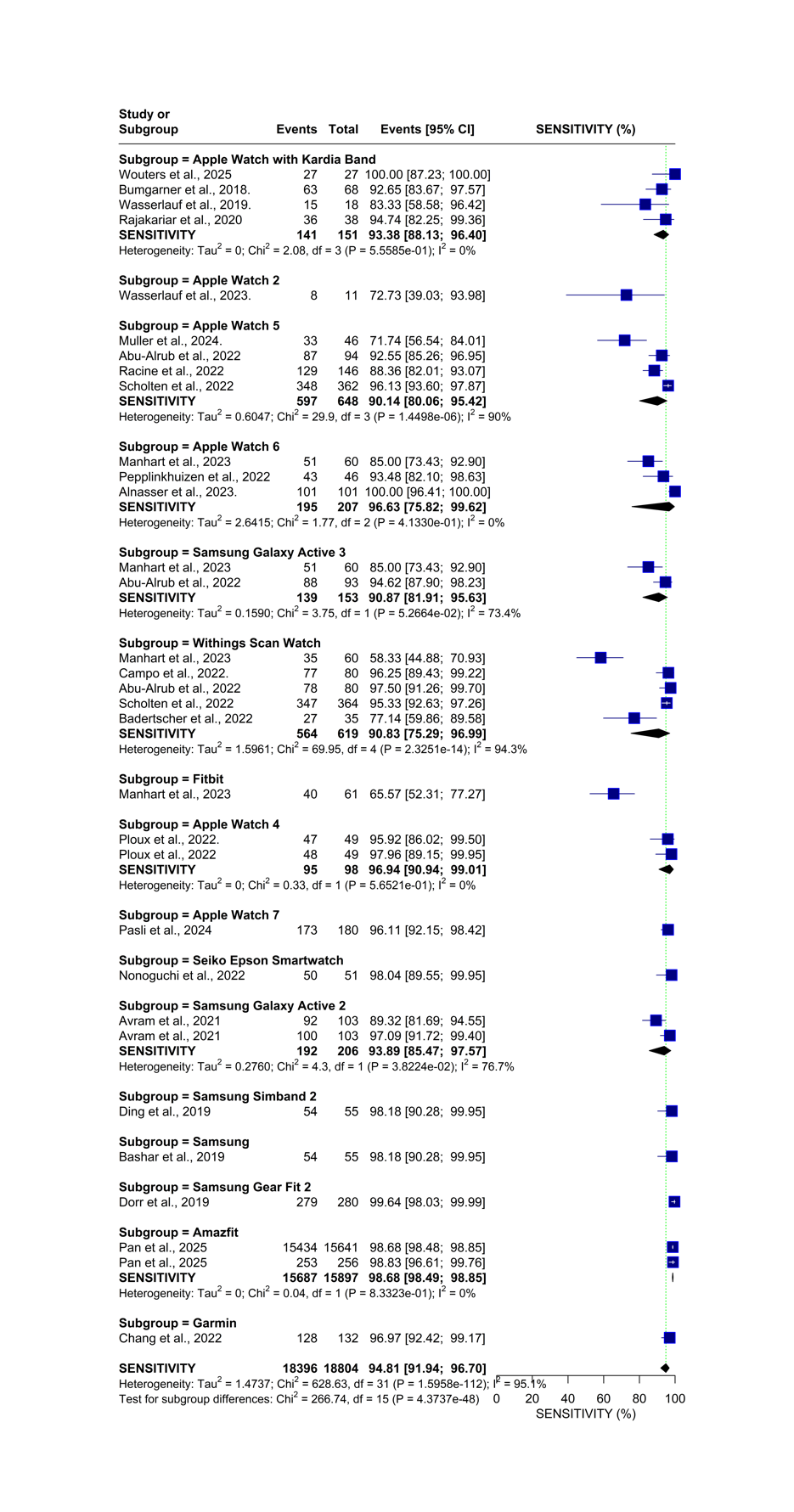


Subgroup analysis stratified by device generation to explore sources of heterogeneity in specificity estimates. A modest reduction in heterogeneity was observed across groups.

**Supplemental Figure 13. Forest Plot of the Pooled Specificity Stratified by Device Generation**


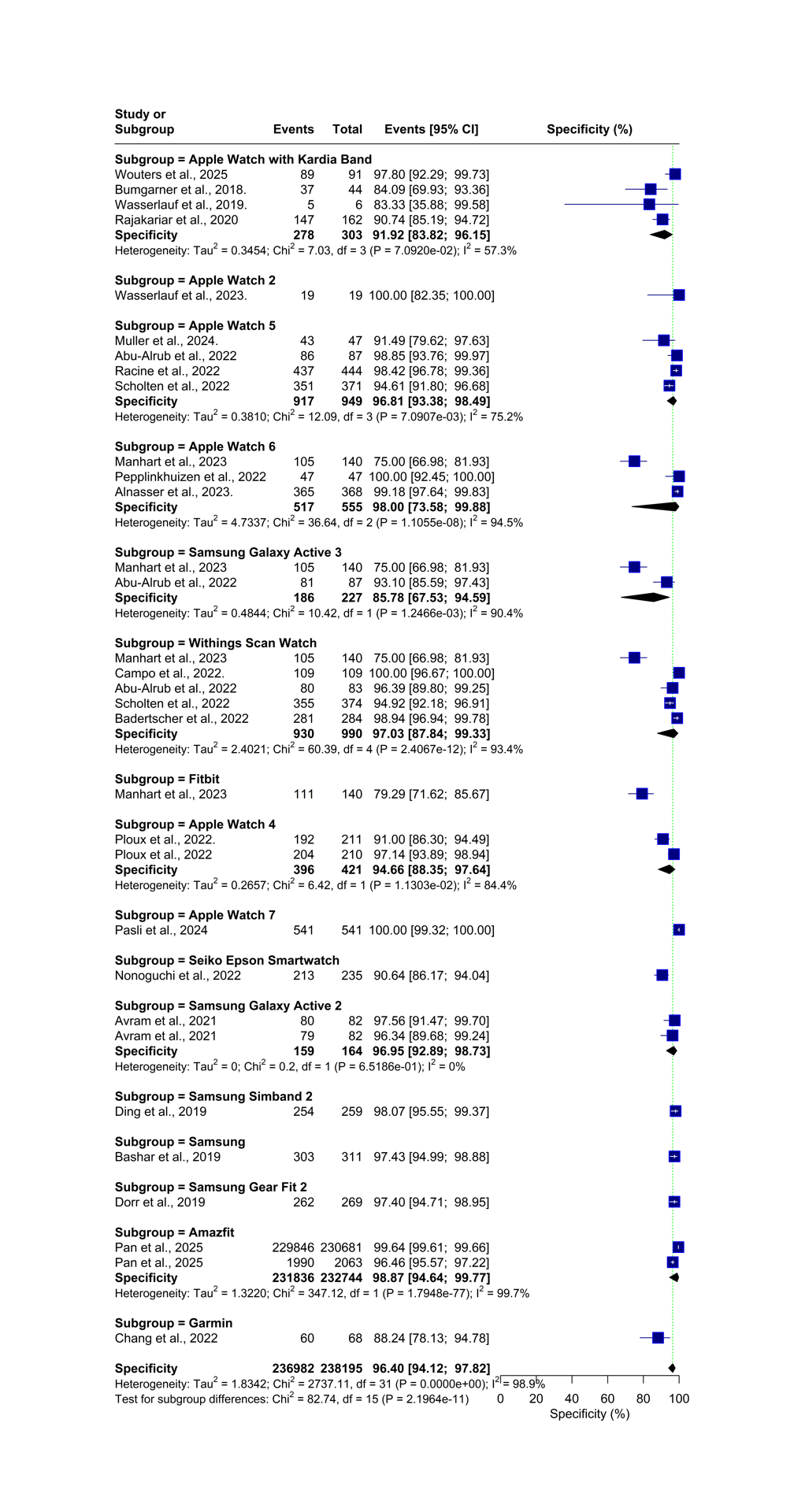


Subgroup analysis stratified by device generation to explore sources of heterogeneity in specificity estimates. A modest reduction in heterogeneity was observed across groups.
